# Register study of migrants’ hospitalization in Norway: world region origin, reason for migration, and length of stay

**DOI:** 10.1186/s12913-016-1561-9

**Published:** 2016-07-26

**Authors:** Jon Ivar Elstad

**Affiliations:** NOVA, Centre for Welfare and Labour Research, Oslo and Akershus University College of Applied Sciences, P.O.B. 4, St. Olavs Plass, 0130 Oslo, Norway

**Keywords:** Hospital care, Utilization, Specialist health services, Country background

## Abstract

**Background:**

The proportion of migrants and refugees increase in many populations. Health planners have to consider how migration will influence demand for health care. This study explores how migrants’ geographical origin, reason for migration, and duration of residence are associated with admission rates to somatic hospitals in Norway.

**Methods:**

Sociodemographic information on all individuals residing in Norway at the start of 2008 was linked to data on all admissions to somatic hospitals during 2008–2011. Migrants, age 30–69, who had come to Norway during 1970–2007 (*N* = 217,907), were classified into seven world region origins and compared with native Norwegians of the same age (*N* = 2,181,948). Any somatic hospital stay 2008–2011 and number of hospital admissions 2008–2011 per 1000 personyears for a set of somatic diagnoses were analyzed by age and gender standardized rates, linear probability models, and Poisson regression.

**Results:**

In the native Norwegian sample, 28.7 % had at least one admission 2008–2011, and there were 116 admissions per 1000 personyears. Corresponding age and gender adjusted figures for the migrant sample were 27.0 % and 103 admissions. Admission rates varied with migrants’ geographical origin, with relatively many admissions among migrants from West and South Asia and relatively few admissions among migrants from Western, East European, and Other Asian countries. Hospitalization varied strongly with reason for migration, with low admission rates for recent work migrants and high admission rates for recent refugees. Admission rates tended to move towards the level among native Norwegians with increasing length of stay. Among longstanding migrants (arrival period 1970–1989), admission rates were close to the levels of native Norwegians for most analyzed migrant categories.

**Conclusion:**

Both world region origin, reason for migration, and duration of residence are important sources for variations in migrants’ utilization of somatic hospitals. Forecasts about migrants’ use of hospital services have to take into account how the migrant population is composed as to these three determinants. High admission rates among recently arrived refugees should be a health policy concern.

**Electronic supplementary material:**

The online version of this article (doi:10.1186/s12913-016-1561-9) contains supplementary material, which is available to authorized users.

## Background

The increasing number of migrants and refugees in many countries is a challenge for the health services. In addition to access and equity issues [1, 2], health politicians will have to ask how the demand for health care is affected. Will the migrants have higher or lower utilization rates than the native populations in the years ahead?

As to hospital care, findings are mixed. A review of European studies, published in 2010, found “contrasting results” [3]. Later analyses from Italy, Netherlands, Germany, Denmark, Spain, and United Kingdom have reported both comparatively high and relatively low use of hospital services among migrants [4–12].

Diverse findings are to be expected, since studies differ in methodology and what type of hospital care they address, and migrant populations vary both between host countries and within each host country over time. There may nevertheless be a set of common factors underlying migrants’ health care utilization, and inconsistent empirical findings could emerge because these factors operate in different contexts. Identifying and analyzing such factors could lead towards an explanatory framework analogous to existing models for migrants’ health [13–15], which could be useful both for understanding the diversities in migrants’ health care utilization and for making forecasts about future demand for health care.

The present study analyzes how world region origin, reason for migration, and length of residence are associated with migrants’ admissions to somatic hospitals in Norway.

Regarding geographical origin, previous research suggests that it may influence migrants’ health care utilization in many ways. Childhood poverty and disease panorama in the home country may have implications for later health [13]. Enabling and predisposing factors for health care use [16], such as health literacy, help-seeking norms, and knowledge about health care providers, may be associated with one’s origin. Country background may furthermore have long term consequences because it affects post-migration life trajectories, for instance labour market participation and risk of social exclusion [17].

As to reason for migration, research indicates that work migrants often have relatively low health care utilization [18]. This is commonly attributed to the so-called healthy migrant effect. Transnational migration will often be strenuous, and those who lack sufficient mental and physical strength may refrain from emigrating [19]. The selection effect may be so strong that migrants’ health is better not only than the average in the population they leave, but also in the population they enter [20]. The healthy migrant effect is likely to be particularly evident among work migrants, since self-selection is dominant and potential migrants are aware that ill health will impede their chances on foreign labour markets. The health-selective mechanisms are probably weaker for family re-unification, and when emigration is forced, more or less, because of war, famine or persecution, both pre-migration conditions and the migration experience itself may be traumatic and causing health problems [21, 22]. Thus, a Danish study found high disease rates among refugees and also, to some extent, among family-united migrants [4], while a Norwegian study observed considerable multimorbidity among refugees, but less among family migrants and even less among work migrants [18].

Accordingly, migrants’ health care utilization is likely to vary both with country origin and reason for migration, but the effects could depend on length of stay in the new country. Previous research has indicated that length of residence is associated both with health status [4, 17, 18, 23–29] and use of health services [30–34]. Low utilization rates are often found among newly arrived migrants, partly because of the healthy migrant effect and partly because of unfamiliarity with the healthcare system in the new country [35]. With increasing duration of stay, migrants’ health care utilization may increase [4, 13, 19, 36]. One reason is acculturation, i.e., how migrants adapt to the dominant culture, its value systems and prevailing lifestyles [11, 37]. As both health-related behaviours and help-seeking norms are affected [38, 39], acculturation could mean that migrants’ disease profiles and utilization patterns gradually approximate and converge with those of the non-migrant population [36, 40–42]. If being exposed to long lasting detrimental environments such as low income, unhealthy working conditions, and discrimination, utilization levels may even surpass non-migrants’ levels. Indications of such processes are the rise in migrants’ multimorbidity with increasing length of stay [18], and the relatively high mortality among guest workers who came to Germany in the 1960s and had “hard working conditions in their lifetimes” [29].

Using Norwegian register data which cover the entire population, the present study explores these issues further. The review above leads towards two expectations: (1) Migrants’ use of somatic hospitals will vary markedly both with country background, reason for migration, and duration of residence, and migrants’ hospitalization rates will, to a considerable extent, be a function of how the migrant population is composed as to these three factors. (2) After a prolonged stay in the host country, a tendency towards convergence will occur, i.e., migrants will have hospitalization rates which do not deviate much from those of the non-migrants, irrespective of country background and the original reason for migration.

The context of the present study is the Norwegian healthcare system, which has some characteristic features. Hospitals are tax funded and, with few exceptions, state owned, and in-patient hospital care is free for all registered inhabitants [43]. Therefore, low income does seldom preclude hospital admissions, but a “pro-rich” bias in utilization of Norwegian specialist health care has nevertheless been found [44]. Hospital admissions are, on the other hand, regulated by the gate-keeping role of General Practitioners (GPs). The patient list system, implemented in 2001, implies that practically all inhabitants are registered with a particular GP (or GP office). Normally, hospitalization will require a referral from the patient’s ordinary GP, or from a physician specialist who has examined the patient on request from the patient’s GP. Thus, admissions to somatic hospitals in Norway depend strongly on physicians’ decisions, but this does not, of course, prevent that also patients’ wishes, preferences, and resources could play a role.

## Methods

### Data, sample, variables

The data file used for this study was constructed by linking sociodemographic information for all registered inhabitants per January 1, 2008, provided by Statistics Norway, with data about the activities of practically all somatic hospitals in Norway, collected by Norwegian Patient Register. Hospitalization during 2008–2011, i.e., admissions to in-patient care in one of the somatic hospitals, intended to last for at least one night, was analyzed for those aged 30–69 at the start of 2008 (ca. 2.4 millions). Those above age 70 were excluded because there were few elderly non-Western migrants in Norway in 2008, and those below age 30 were excluded because they have few hospital admissions (except for admissions related to childbirths).

Two outcome variables were analyzed. The first is a dichotomy indicating whether the individual had at least one admission to a somatic hospital during the 4 years 2008–2011. This outcome variable gives an overall indication of hospital utilization, but it does not distinguish between few and many hospital stays and includes admissions without clear links to diagnosed disease (e.g., diffuse symptoms, routine check-ups, normal childbirths). Therefore, also a second outcome variable was analyzed: number of admissions during 2008–2011 due to tumours (neoplasms) and conditions of the circulatory, musculoskeletal, digestive, respiratory, and endocrine systems, and some other smaller somatic diagnostic categories, i.e., Chapters I to IV and VI to XIV in the International Classification of Diseases (ICD), 10th Revision [45]. The second outcome variable included more than 60 % of all admissions for the 30–69 age categories. The distribution of admissions on major diagnosis categories is shown in Additional file 1: Table S1.

For this study, migrants were defined as individuals who, according to the population register, were born abroad by two non-Norwegian parents. Migrants with immigration year 1970–2007 (*N* = 217,907) were analyzed and compared with all other inhabitants, here termed non-migrants or native Norwegians (*N* = 2,181,948). Pre-1970 migrants were excluded since earlier migration to Norway was rare and primarily from neighbouring Nordic countries.

Due to data protection stipulations, information about migrants’ country background was only available in terms of 18 categories, either specific countries (e.g., Sweden, Poland, Vietnam) or groups of countries (e.g., West Europe, Africa apart from Somalia, Latin America). This information was recoded into seven world region origins. Migrants from Nordic, West European, and overseas Western countries were pooled as Western migrants. Central and East European categories were divided between current European Union (EU) members countries and Other East Europe. The two African categories, Somalia and Other Africa, were combined. Migrants from Turkey, Iraq, Iran, Pakistan, and Sri Lanka, were pooled into a West and South Asia category. Remaining Asian countries, mostly in East Asia, were termed Other Asia. The original Latin America category was retained.

The immigration authorities began to collect information about reason for migration in 1990. As migration between Nordic countries has been practically unrestricted for decades, reason for migration from neighbouring Sweden, Denmark, Finland and Iceland is almost never recorded. Migrants 1990–2007 were classified either as work migrants (including a few who came for education), family re-unification, refugees, or unknown – the latter category was used for all pre-1990 migrants and for later migrants with missing information.

Migrants’ length of stay was indicated by grouping migration year into five periods, from recent migrants (arrival years 2005–2007) to longstanding migrants (1970–1989).

Other variables used in the analyses were age (at the start of 2008) and gender. Due to data protection considerations, age information was only available in 10-years bands; 30–39 years, 40–49 years, 50–59 years and 60–69 years. Personyears 2008–2011 were calculated for each individual by means of information given in the data file about deaths and emigration during 2008–2011. Missing values on the variables used in this study were negligible.

### Analyses

After describing the samples, variations in hospitalization within the migrant sample according to reason for migration, world region origin, and length of stay, were examined. This analysis was restricted to migrants who came during the 1990–2007 period, since no information about reason for migration was available for earlier migrants. Estimations of percentages with at least one somatic hospital admission 2008–2011, men and women together, were made, directly standardized with age and gender composition of the native Norwegian comparison sample, age 30–69, as standard population. Likewise, number of somatic hospital admissions 2008–2011 per 1000 personyears, age and gender standardized, for the selection of somatic diagnoses described above, were calculated.

Next, the convergence issue was examined by analyzing how recent and longstanding migrants’ hospitalization rates deviated from the rates among non-migrant native Norwegians. In gender stratified analyses adjusted for age, migrants with registered migration in 2005–2007 were compared with migrants who were registered during 1970–1989. Non-migrant native Norwegian men and women, respectively, were used as reference categories in these analyses. The outcome any hospital admission 2008–2011 was analyzed by linear probability models, i.e., OLS linear regression, while number of admissions 2008–2011 for the selected set of diagnoses was analyzed by Poisson regression, with individuals’ personyears 2008–2011 as exposure. Analyses were made with STATA Release 13, programs *tab*, *dstdize*, *reg*, and *poisson*, and statistical significance was assessed by robust standard errors [46, 47].

## Results

The sample description (Table 1) demonstrates how migration to Norway since the 1970s had generated a very heterogeneous migrant population in 2008. The long-established migration from Nordic and a few other Western countries continued, but migration from other parts of the world has changed the composition of the migrant population since the 1970s. Work migrants from Pakistan, Turkey, and partly from North Africa came already during the 1970s, as well as refugees from Latin America. In the subsequent decades, non-Western migrants were mostly refugees or family migrants, many of them from Vietnam, Iran, the Balkans, Somalia, Eritrea, and Iraq later on. Table 1 indicates that migration due to family unification has been substantial. In the mid-2000s (2005–2007), work migration increased much, in particular from East Europe, typically Poland, but also from the Nordic countries.

**Table 1:** Description, samples of migrants 1970–2007 and non-migrant native Norwegians, age 30–69 per January 1, 2008

World region origin	Total N	Immigration period (number of individuals)					Reason for migration (number of individuals)				Hospitalization 2008–2011, standardized^a^	
		2005–2007	2001–2004	1996–2000	1990–1995	1970–1989	Work	Family	Refugee	Un-known	Any admission (%)	Admissions per 1000 personyears
Western countries	62,184	10,464	8704	12,114	7989	22,913	11,643	7169	122	43,250	25.3	94
EU East Europe	24,443	15,015	2900	1636	1762	3130	15,295	4768	190	4190	21.9	80
Other East Europe	23,382	2382	4457	5285	9032	2226	1061	6291	12,811	3219	28.7	103
Africa	22,280	3348	5105	4411	3568	5848	477	6317	8228	7258	29.0	110
West & South Asia	39,633	1987	5162	8741	6519	17,224	414	10,798	10,184	18,237	32.5	137
Other Asia	37,139	5524	7110	4517	6065	13,923	1683	13,616	6052	15,788	24.6	92
Latin America	8846	1008	1226	1254	931	4427	379	3357	277	4833	29.6	101
Total	217,907	39,728	34,664	37,958	35,866	69,691	30,952	52,316	37,864	96,775	27.0	103
Number, sample of non-migrant native Norwegians, age 30–69: 2,181,948											28.7	116

^a^Directly age and gender standardized using native Norwegians, age 30–69, per January 1, 2008, as standard population

As to the central topic in this study, the two right-hand columns in Table 1 are especially relevant. They show that among native Norwegians, age 30–69, men and women pooled, 28.7 % had at least one admission to a somatic hospital during 2008–2011, and there were 116 somatic hospital admissions per 1000 personyears for the selected diagnoses. Because of large differences in age and gender compositions, standardization is necessary for making meaningful comparisons between migrants and non-migrants, and between migrants with different geographical origins. Table 1 shows that after standardizing, the migrant population had a lower percentage with at least one hospitalization than the non-migrant native Norwegians (27.0 % versus 28.7 %). They also had fewer admissions per 1000 personyears (103 versus 116). Table 1 indicates furthermore that hospitalization rates varied much with background – percentages with at least one admission ranged from 21.9 % (migrants from EU East Europe) to 32.5 % (West and South Asia), and number of admissions per 1000 personyears varied from 80 to 137 between these two world region origins.

**Fig. 1:**
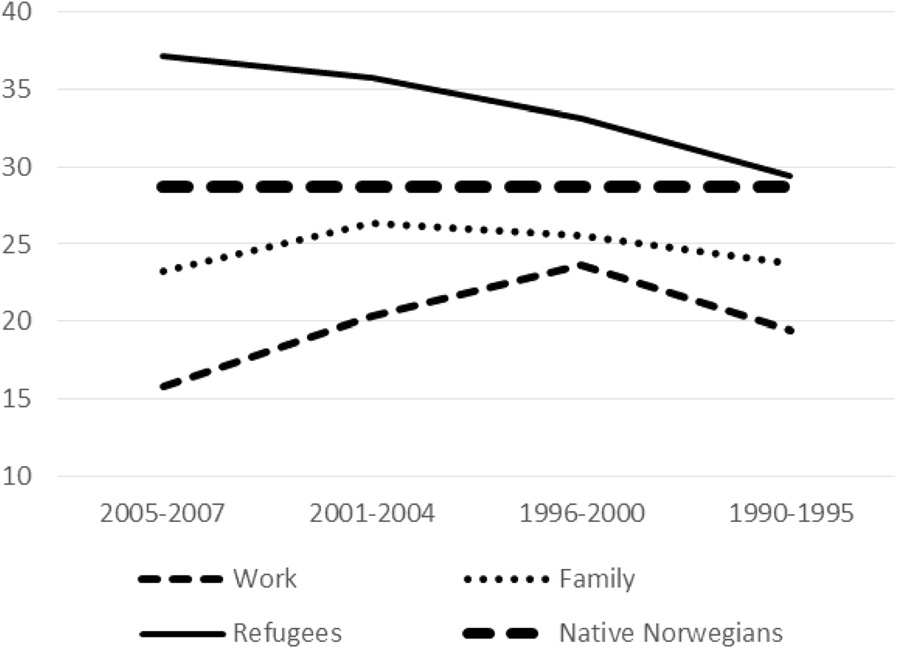
Any hospitalization 2008–2011 (%), age and gender standardized, native Norwegians and migrants by immigration period and reason for migration

**Fig. 2:**
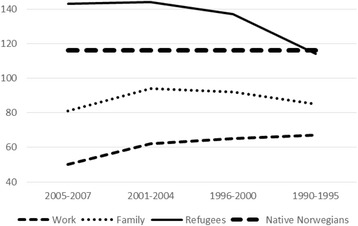
Admissions per 1000 personyears 2008–2011 (%), age and gender standardized, native Norwegians and migrants by immigration period and reason for migration

Table 2 shows how hospitalization rates among the migrants were simultaneously patterned by reason for migration, world region origin, and length of stay. Estimations were standardized for age and gender in order to make rates comparable to those of the native Norwegians. Only migrants who came 1990–2007 were included in this analysis, since information about reason for migration lacked for pre-1990 migrants. Small categories, such as refugees from EU East Europe and work migrants from Africa, were pooled into larger categories. Confidence intervals were not reported in order to avoid overloading the table, but the few estimations made on subsamples with less than 200 individuals were enclosed in parentheses. The main results of Table 2 are also displayed in Figs. 1 and 2.

**Table 2:** Migrants’ hospitalization 2008–2011 by reason for migration, world region origin, and immigration period

		Any hospital admission (%), age and gender standardized^a^					Number of admissions per 1000 personyears, age and gender standardized^a^				
Reason for migration	Immigration period/region origin	All periods	2005–2007	2001–2004	1996–2000	1990–1995	All periods	2005–2007	2001–2004	1996–2000	1990–1995
Work	Western countries	21.2	19.0	20.7	23.0	23.4	66	71	68	63	68
	EU East Europe	14.8	13.7	24.0	(20.6)	(17.0)	39	37	55	(49)	(74)
	Other countries	19.3	11.9	17.3	26.6	18.3	55	29	53	78	67
	All region origins	19.2	15.8	20.3	23.6	19.4	56	50	62	65	67
Family	Western countries	22.2	20.6	24.2	21.7	20.7	76	70	82	75	76
	EU East Europe	23.4	20.6	19.8	25.5	24.5	96	113	68	100	110
	Other East Europe	24.7	26.0	24.2	26.8	20.6	71	63	74	72	65
	Africa	29.1	36.4	25.2	28.1	29.8	101	136	109	95	99
	West and South Asia	29.3	29.3	32.5	29.0	27.8	113	97	106	128	107
	Other Asia	22.1	19.2	23.9	22.6	21.8	84	71	111	93	75
	Latin America	27.5	26.3	25.9	25.2	29.7	82	75	61	77	97
	All region origins	24.9	23.2	26.3	25.5	23.7	88	81	94	92	85
Refugees	Other East Europe	30.6	40.1	33.6	30.4	29.6	117	150	119	126	112
	Africa	32.5	36.4	33.9	28.7	31.8	120	133	129	98	117
	West and South Asia	36.6	40.0	40.4	37.5	32.3	160	140	175	164	146
	Other Asia	30.1	36.1	33.4	31.2	24.6	120	147	141	131	91
	All region origins	32.4	37.1	35.7	33.1	29.4	127	143	144	137	114
Unknown	All region origins	27.1	23.2	26.6	26.9	26.1	104	101	109	108	98
Total	All region origins	26.4	22.1	28.2	28.2	26.7	100	79	110	111	100

^a^Age and gender standardization for each immigration period and each region origin. Standard population = native Norwegians, age 30–69, per 1 January 2008. Estimations based on subsamples with less than 200 individuals in parentheses

Large differences in standardized rates according to reason for migration can be observed. On average, 19.2 % of the work migrants, 24.9 % of the family unification migrants, and 32.4 % of the refugees, had at least one admission. The corresponding number of hospital admissions per 1000 personyears were 56, 88, and 127, respectively.

Moreover, hospitalization varied with length of stay. Among recent refugees (arrival years 2005–2007), all regions together, 37.1 % had at least one admission, but among refugees who arrived in 1990–1995, the percentage was 29.4 %, i.e., close to the “native” level of 28.7 %. Recent work migrants had low hospitalization rates (average all regions: 15.8 % with at least one admission, 50 admissions per 1000 personyears). Work migrants with longer stays in Norway had somewhat higher hospitalization levels. For family unification migrants, however, hospitalization rates did not vary much with migration period.

Thus, length of stay, and reason for migration even more, appear to be strongly associated with variations in migrants’ admission rates to somatic hospitals. Within migrant categories defined by reason for migration and length of stay, there were sometimes marked differences according to geographical origin. Among the 2005–2007 family unification migrants, for instance, percentages with at least one hospital admission varied from 19.2 % (Other Asia) to 36.4 % (Africa). Nevertheless, Table 2 also suggests some commonalities. Whatever their world region origin, recent work migrants had low hospitalization rates, while longer stays were associated with higher admission rates. Likewise, high hospitalization rates were observed among recent refugees, but hospitalization levels decreased consistently with increasing length of stay, whatever part of the world the refugees came from.

Lastly, hospitalization rates for recent and longstanding migrants, relative to native Norwegians, were analyzed separately for men (Table 3) and women (Table 4) – all migrants pooled, and for each of the seven world region origins, adjusted for age.

**Table 3:** Hospitalization among recent and longstanding male migrants, relative to native Norwegian men

	Any hospital admission 2008–2011, linear probability models				Number of hospital admissions 2008–2011, Poisson regression models			
Immigration period	2005–2007		1970–1989		2005–2007		1970–1989	
	B	95%CI	B	95%CI	IRR	95%CI	IRR	95%CI
Constant/reference	0.298	0.296/0.299	0.298	0.297/0.300	1		1	
Migrants all regions	−0.079	−0.083/−0.075	−0.012	−0.016/−0.007	0.601	0.566/0.639	0.944	0.916/0.974
Migrants classified by geographical origin								
Western countries	−0.072	−0.080/−0.064	−0.020	−0.028/−0.012	0.615	0.554/0.682	0.886	0.841/0.933
EU East Europe	−0.104	−0.109/−0.099	−0.010	−0.034/0.015	0.436	0.393/0.485	0.988	0.838/1.165
Other East Europe	−0.040	−0.064/−0.015	−0.011	−0.035/0.012	0.818	0.646/1.037	0.840	0.717/0.984
Africa	−0.019	−0.036/−0.002	−0.046	−0.058/−0.034	1.061	0.882/1.275	0.795	0.715/884
West & South Asia	−0.008	−0.032/0.016	0.025	0.017/0.033	0.812	0.661/0.998	1.172	1.109/1.238
Other Asia	−0.056	−0.071/−0.041	−0.041	−0.051/−0.032	0.944	0.786/1.133	0.832	0.769/0.900
Latin America	−0.050	−0.087/−0.013	0.002	−0.016/0.020	0.650	0.436/0.969	0.894	0.795/1.007
R^2^/Pseudo R^2^	0.043		0.041		0.066		0.064	
N native Norwegians	1,102,919		1,102,919		1,102,919		1,102,919	
N migrants	25,718		38,052		25,718		38,052	

Adjusted for four age categories: 30–39, 40–49, 50–59, and 60–69, reference category = 50–59, age coefficients not shown. B = OLS regression coefficient. *IRR* incidence rate ratio. Exposure variable, Poisson regression: personyears

**Table 4:** Hospitalization among recent and longstanding female migrants, relative to native Norwegian women

	Any hospital admission 2008–2011, linear probability models				Number of hospital admissions 2008–2011, Poisson regression models			
Immigration period	2005–2007		1970–1989		2005–2007		1970–1989	
	B	95%CI	B	95%CI	IRR	95%CI	IRR	95%CI
Constant/reference	0.278	0.277/0.280	0.278	0.277/0.280	1		1	
Migrants all regions	0.021	0.013/0.029	−0.009	−0.014/−0.004	0.778	0.731/0.829	0.926	0.894/0.958
Migrants classified by region origin								
Western countries	−0.000	−0.015/0.014	−0.036	−0.044/−0.028	0.771	0.667/0.891	0.812	0.765/0.862
EU East Europe	−0.057	−0.074/−0.039	−0.029	−0.049/−0.009	0.517	0.437/0.611	0.840	0.723/0.977
Other East Europe	0.044	0.020/0.068	0.016	−0.014/0.046	0.742	0.617/0.893	1.149	0.936/1.409
Africa	0.131	0.105/0.157	0.009	−0.012/0.030	1.193	1.035/1.374	1.007	0.873/1.161
West & South Asia	0.130	0.099/0.161	0.046	0.034/0.058	1.036	0.867/1.238	1.256	1.175/1343
Other Asia	0.004	−0.011/0.020	−0.040	−0.050/−0.030	0.744	0.654/0.847	0.769	0.711/0.832
Latin America	0.054	0.018/0.091	0.060	0.040/0.081	0.747	0.584/0.956	1.102	0.984/1.234
R^2^	0.019		0.019		0.037		0.036	
N native Norwegians	1,079,029		1,079,029		1,079,029		1,079,029	
N migrants	14,010		31,639		14,010		31,639	

Adjusted for four age categories: 30–39, 40–49, 50–59, and 60–69, reference category = 50–59, age coefficients not shown. B = OLS regression coefficient. *IRR* incidence rate ratio. Exposure variable, Poisson regression: personyears

Table 3 shows that recent male migrants (arrival 2005–2007), all origins combined, had markedly less hospitalization than native Norwegian men. The linear probability model coefficient, minus 0.079, implies that the age-adjusted percentage with at least one somatic hospital admission was 7.9 percentage points lower among migrant men than among native Norwegian men. Similarly, the incidence rate ratio (IRR) in the corresponding Poisson regression model for recent migrant men was 0.601, i.e., far below unity, indicating that also the age-adjusted number of hospital admissions for the selected diagnostic categories were much lower among recent migrant men than among native Norwegian men.

In contrast, the analyses in Table 3 of longstanding migrant men (arrival years 1970–1989) show that they, overall, had hospitalization rates close to those of native Norwegian men. The linear probability regression coefficient was only minus 0.012, while the IRR was 0.944. Both coefficients were significantly lower than the native level (cf. the 95 % confidence intervals), but the size of the coefficients indicate that admission rates to somatic hospitals differed little between longstanding male migrants and non-migrant men.

The main pattern – much lower hospitalization among recent migrants and very small differences for longstanding migrants – emerged also for male migrants from Western, East European, and Latin American countries. A different pattern, although in line with the assumption of increasing utilization with increasing length of stay, can be seen for male migrants from West and South Asia – compared to native Norwegian men, recent male migrants from these countries had similar hospitalization levels, but longstanding migrants had clearly higher hospitalization rates. Among male migrants from Africa and Other Asia, hospitalization patterns varied in a more irregular way.

The results from corresponding analyses among female migrants are shown in Table 4. Low hospitalization rates among recent female migrants, and similar and sometimes higher rates among longstanding female migrants compared to native Norwegian women, emerged also in the Poisson regression analyses of number of hospital admissions (Table 4, right part). The results from the linear probability models were somewhat different, however, since the overall proportion who had at least one somatic hospital admission 2008–2011 was somewhat higher (+0.021, i.e., 2.1 percentage points) among recent female migrants than among native Norwegian women. The explanation for the discrepancy between the Poisson and linear probability models when analyzing women is probably that when any hospital admission is analyzed (the linear probability models), admissions due to normal childbirths and vague and undiagnosed symptoms are included. The Poisson analyses of number of admissions include only somatic disease diagnoses. A higher number of childbirths are therefore likely to be some of the reason why recently arrived female migrants, more often than native Norwegian women, had at least one hospital admission (cf. the large positive coefficients, around plus 0.13, for recent migrant women from Africa and West and South Asia).

## Discussion

### Main results

This study has shown that overall, the migrant population in Norway, age 30–69, had lower (age and gender adjusted) admission rates to somatic hospitals during 2008–2011 than the native Norwegian population. This occurred for both outcomes analyzed here: at least one admission (27.0 % versus 28.7 %), as well as number of hospital admissions due to a set of somatic diagnoses (103 versus 116 per 1000 personyears).

The main objective of the present study was to examine how migrants’ hospitalization levels were conditioned by country background (measured in this study by seven world regions), reason for migration, and length of stay in Norway.

Distinct differences in hospitalization according to reason for migration emerged. Overall, age and gender adjusted hospitalization rates among refugees were around twice as high as the rates among work migrants, while family unification migrants had overall somewhat lower hospitalization rates than native Norwegians. This concurs with findings in previous studies [4, 18, 34] and with the expectation that the healthy migrant effect is particularly prevalent among work migrants, but often absent among refugees who could have been exposed to many types of health risks in their pre-migration lives [19, 48, 49].

Variations in hospitalization rates according to geographical origin were also marked. Relatively high admission rates – often higher than among native Norwegians – were observed among migrants with a background from West and South Asian countries. Relatively low rates were found among migrants from the European Union member countries in East Europe and also among migrants from Western and Other Asian countries.

Interestingly, the analyses suggested that the differences between world region origins were strongly associated with reason for migration. Among the migrants who came from West and South Asian countries during 1990–2007, there were more than 10,000 refugees, but less than 500 work migrants. The high hospitalization rates among migrants from these countries were probably due, at least partly, to a large proportion of refugees. Another indication of the impact of reason for migration is the finding that work migrants both from non-Western and Western countries had low hospitalization rates.

However, hospitalization differences according to reason for migration seemed to diminish with increasing length of stay. Among recent migrants (arrival years 2005–2007), 15.8 % of the work migrants and 37.1 % of the refugees had at least one hospital admission, but this difference was clearly smaller (19.4 % versus 29.4 %) among those who came during 1990–1995 and therefore had lived in Norway for at least 13 years. Hospitalization rates increased with increasing duration of stay for work migrants, but decreased for refugees. The reason that rates declined among refugees could be that they “benefit from improvements in health care, hygiene and nutritional conditions” [13] and from relief from fear and strain, resulting in decreasing need for health care. Increasing rates among work migrants could occur if the healthy migrant effect “wears off” and work life hazards begin to play a role.

Thus, in line with previous studies on health care utilization [30–34], this study reiterates the role of length of stay. This factor may have been underrated because of missing information; the 2010 review of European studies of migrants’ health care utilization noted that only one of 21 reviewed studies had information about time in the host country [3].

Whether migrants’ health care utilization patterns will converge towards the native level over time is a current debate [36, 42]. Among longstanding migrants in this study (arrival years 1970–1989), hospitalization rates 2008–2011 were quite close to the rates among native Norwegians for migrants from several world regions. This suggests convergent utilization levels when living in the new country for two decades or more. However, other factors will influence the associations between utilization levels and length of stay. The large proportion with at least one hospital admission among women from Africa and West and South Asia with a short stay in Norway was probably due to frequent childbirths, while relatively high hospitalization rates among pre-1990 male and female migrants from South and West Asia could be partly due to harsh living conditions during their years in Norway.

### Policy implications

The role of country background and geographical origin for migrants’ use of health care have for long been acknowledged. The present study underlines that also reason for migration and length of stay are important sources for variations in health care use. Migrants’ utilization levels at any time point are likely to be conditioned, to a considerable extent, by the existing composition of the migrant population as to these factors. If recent work migrants constitute a large proportion of the migrant population, overall hospital utilization is likely to be markedly lower than that of the non-migrant population. If, on the other hand, work migration has dwindled and a large proportion of the migrant population are either newly arrived refugees or longstanding migrants who have encountered many environmental hazards in the host country, the migrant population will probably use health care more than non-migrant natives.

This implies that predictions about the future utilization of somatic hospitals in the migrant population will be uncertain, since utilization levels will depend on several unknowns such as the number of new migrants in the future, their reasons for migration, and their country background. In addition, not only country background, reason for migration, and length of stay, but also other factors such as migrants’ educational level and their typical social trajectories in the host country will influence their utilization rates.

The findings of this study suggest furthermore that the health services should pay particular attention to two categories of migrants. Firstly, the newly arrived refugees will often be a particularly vulnerable category, often in need of culturally sensitive health care – both somatic hospital services and mental health care [22]. Secondly, healthy work migrants may experience a relatively fast health deterioration [18], which could be due to difficult working conditions, low material level of living, and social isolation. One task for the health services could be to develop appropriate preventative services for this migrant category.

### Study strengths and limitations

One strength of this study is that it examines unselected samples comprising all registered migrants in Norway, aged 30–69 as of January 1, 2008. Another strength is that these registers are known for high quality and good updating routines. Since the Norwegian Patient Register collects information from practically all somatic hospitals in Norway (except for a few, minor, private hospitals), the estimated hospitalization rates are probably quite precise.

However, a definite weakness is the very heterogeneous geographical origin categories used in this study. It is not unlikely that migrants from the different countries pooled into the seven world origins differ considerable in admission rates to somatic hospitals. This could hardly be avoided with the available data, however. Specific country background was available for some migrant origins (e.g., Sweden, Poland, Vietnam), but for the majority of the migrants, information was only available in terms of broad categories such as Nordic countries other than Sweden, Western Europe, Africa outside Somalia, and Latin America.

Another limitation in this study is that it only refers to registered inhabitants and gives no information about use of hospital services among undocumented migrants.

Moreover, only admissions to somatic hospitals have been addressed. Research indicates that mental health problems could be widespread among some migrant categories, and among refugees in particular [22, 50]. This implies that also migrants’ admissions to psychiatric hospitals and mental health institutions should be examined in order to obtain a more comprehensive picture of migrants’ utilization of hospital services.

A very important interpretational difficulty should be noticed. The observed associations between hospitalization rates and length of stay could be due not only to the effects of increasing duration of residence, for instance because of acculturation processes or exposures during migrants’ life courses in Norway. Another possibility is that various circumstances have differed considerably between the migrants who came during, say, the early 1990s, and those who arrived in the mid-2000s. The observed associations with length of stay could, more or less, reflect variations in a number of health-related characteristics between different cohorts of migrants. This interpretational difficulty makes conclusions about how length of stay affects hospitalization somewhat uncertain.

As to methods, one may question that a dichotomous outcome (i.e., any hospital admission 2008–2011) was analyzed by linear probability models (Tables 3 and 4). For such outcomes, logistic models are more common. Linear probability models were chosen since the coefficients, indicating differences in proportions, are more easy to interpret than logit estimates and odds ratios. Moreover, results from logistic and linear probability models do seldom lead to different interpretations [51]. For completeness, Additional file 1: Table S2 gives the results from logistic regression analyses of the any hospital admission outcome.

## Conclusions

Overall, the migrant population in Norway in 2008, aged 30–69, had lower age and gender standardized utilization of somatic hospitals during 2008–2011 than the non-migrant native population. Variations with migrants’ geographical origin were considerable. Migrants from West and South Asia had higher hospitalization rates than native Norwegians, while relatively low utilization levels were observed among migrants from Western and European Union countries, as well as among migrants from other parts of Asia. Differences in migrants’ hospitalization rates according to their geographical origin were however strongly associated with reason for migration, as well as influenced by duration of residence in Norway. Work migrants tended to have few hospital admissions during the first years, but higher utilization levels after living for some years in their new country. Recent refugees had, on the other hand, high hospitalization rates, but refugees who came in the early 1990s had age and gender adjusted hospitalization rates similar to the native population. Among longstanding migrants who had arrived before 1990, hospitalization rates were often quite close to the level among non-migrant native Norwegians, suggesting a convergence tendency when migrants had lived for two decades of more in the host country. In general, hospitalization levels were strongly conditioned by the composition of the migrant population as to country and geographical background, reason for migration, and length of stay.

## Abbreviations

B, unstandardized OLS regression coefficients; EU, European Union; GP, general practitioner; ICD, international classification of diseases; IRR, incidence rate ratio; OLS, ordinary least square

## Additional file

Additional file 1: Table S1. Admissions (%) 2008–2011 by ICD main chapters, migrants and non-migrant native Norwegians, age 30–69. **Table S2.** Logistic regression models, outcome any hospital admission 2008–2011, reference category native Norwegians, age adjusted*. (DOC 52 kb)
