# Supplementary material for: Register study of migrants’ hospitalization in Norway: world region origin, reason for migration, and length of stay
Source: BMC Health Serv Res. 2016 Jul 26;16:306. doi: 10.1186/s12913-016-1561-9 (PMC4962451; doi:10.1186/s12913-016-1561-9)
Supplement: Additional file 1: Table S1. — Admissions (%) 2008–2011 by ICD main chapters, migrants and non-migrant native Norwegians, age 30–69. Table S2. Logistic regression models, outcome any hospital admission 2008–2011, reference category native Norwegians, age adjusted*. (DOC 52 kb) [file 12913_2016_1561_MOESM1_ESM.doc]

**Additional file 1: Table S1. Admissions (%) 2008-2011 by ICD main chapters, migrants and non-migrant native Norwegians, age 30-69**

| ICD chapter | Label | Migrants | Native Norwegians |
| --- | --- | --- | --- |
|  |  |  |  |
| II | Neoplasms | 8.5 | 12.3 |
| IV | Endocrine | 1.9 | 1.9 |
| IX | Circulatory | 13.0 | 15.4 |
| X | Respiratory | 4.6 | 6.3 |
| XI | Digestive | 7.5 | 7.7 |
| XII | Musculoskeletal | 7.3 | 8.7 |
| I+III+VI-VIII+XII+XIV | Other diseases | 17.9 | 18.4 |
| Other ICD chapters |  | 39.1 | 29.3 |
| Total |  | 100.0 | 100.0 |
|  |  |  |  |
| N all admissions |  | 109,004 | 1,413,808 |
|  |  |  |  |

**Additional file 1: Table S2. Logistic regression models, outcome any hospital admission 2008-2011, reference category native Norwegians, age adjusted***

|  | Men | | | | |  | Women | | | | |
| --- | --- | --- | --- | --- | --- | --- | --- | --- | --- | --- | --- |
| Immigration period | 2005-2007 | |  | 1970-1989 | |  | 2005-2007 | |  | 1970-1989 | |
|  | OR | p-val |  | OR | p-val |  | OR | p-val |  | OR. | p-val |
|  |  |  |  |  |  |  |  |  |  |  |  |
| Native Norw. (ref) | 1 |  |  | 1 |  |  | 1 |  |  | 1 |  |
|  |  |  |  |  |  |  |  |  |  |  |  |
| Western countries | 0.588 | <0.001 |  | 0.903 | <0.001 |  | 0.998 | 0.955 |  | 0.830 | <0.001 |
| EU East Europe | 0.423 | <0.001 |  | 0.950 | 0.430 |  | 0.759 | <0.001 |  | 0.862 | 0.005 |
| Other East Europe | 0.765 | 0.003 |  | 0.940 | 0.344 |  | 1.221 | <0.001 |  | 1.083 | 0.282 |
| Africa | 0.880 | 0.036 |  | 0.764 | <0.001 |  | 1.744 | <0.001 |  | 1.046 | 0.386 |
| West & South Asia | 0.950 | 0.532 |  | 1.143 | <0.001 |  | 1.746 | <0.001 |  | 1.244 | <0.001 |
| Other Asia | 0.666 | <0.001 |  | 0.786 | <0.001 |  | 1.020 | 0.591 |  | 0.813 | <0.001 |
| Latin America | 0.696 | 0.020 |  | 1.010 | 0.834 |  | 1.271 | 0.003 |  | 1.329 | <0.001 |
|  |  |  |  |  |  |  |  |  |  |  |  |
| Pseudo R^2^ | 0.037 | |  | 0.035 | |  | 0.016 | |  | 0.015 | |

* Adjusted for four age categories: 30-39, 40-49, 50-59, and 60-69, reference category = 50-59. OR = odds ratio.
